# Supplementary material for: Structural interactions of BWC0977 with Klebsiella pneumoniae topoisomerase IV and biochemical basis of its broad-spectrum activity
Source: Commun Biol. 2025 Nov 25;8:1666. doi: 10.1038/s42003-025-09055-y (PMC12647645; doi:10.1038/s42003-025-09055-y)
Supplement: Supplementary file 2 — Supplementary information [file 42003_2025_9055_MOESM2_ESM.pdf]

Supplementary information for

Structural interactions of BWC0977 with *Klebsiella pneumoniae* topoisomerase IV and biochemical basis of its broad-spectrum activity

Radha Nandishaiah[1,4], Satoshi Murakami[2]*, Shahul Hameed P[1], Maho Aoki[2], Ui Okada[2], Eiki Yamashita[3], Suryanarayanan Venkatesan[1], Nagakumar Bharatham[1], Sudipta Sarma[1], Anirudh P Shanbhag[1], Sreevalli Sharma[1], Ranga Rao[1], Vasanthi Ramachandran[1], Balasubramanian V[1], Santanu Datta[1], Nainesh Katagihallimath[1]*

1. Bugworks Research India Pvt. Ltd. Center for Cellular & Molecular Platforms, National Center for Biological Sciences, GKVK Campus, Bellary Road, Bangalore 560065, India.
2. Department of Life Science and Technology, Institute of Science Tokyo, Yokohama 226-8501, Japan
3. Institute for Protein Research, Osaka University, Suita, Osaka, 565-0871, Japan
4. The University of Trans-Disciplinary Health Sciences and Technology (TDU), Jarakabande Kaval, Bengaluru, Karnataka 560064, India.

*Corresponding authors: Nainesh Katagihallimath, Satoshi Murakami
Email: nainesh@bugworksresearch.com, murakami@life.isct.ac.jp

This PDF file includes:

Supplementary Fig. 1 - 7
Supplementary Table 1 - 5
Uncropped gel images
SI References

Contents

Supplementary Fig 1. BWC0977 inhibition on *E. coli*, *P. aeruginosa* & *S. aureus* gyrase and topoisomerase IV enzyme activities. 3

Supplementary Fig 2. Ciprofloxacin induces double-strand DNA breaks mediated by gyrase and topoisomerase IV enzymes. 4

Supplementary Fig 3. Electron density maps from the 3.05 Å BWC0977 complex. 5

Supplementary Fig 4. Binding mode of ciprofloxacin, moxifloxacin, zoliflodacin and NBTIs shown with gyrase and topoisomerase IV models. 6

Supplementary Fig 5. Structural comparison of ParEC-core complex with doubly nicked DNA duplex and compound 34. 7

Supplementary Fig 6: Fitness of *E. coli* MG1655 WT and target mutant strains treated with BWC0977 in agar MIC experiment. 8

Supplementary Fig 7. Conservation of BWC0977 binding pocket (GyrA and ParC) residues. 9

Supplementary Table 1. Structural comparison among the four ParEC-core complexes in the two functional dimers in the asymmetric unit. 10

Supplementary Table 2. BWC0977 interactions important for topoisomerase IV (ParEC) inhibition. 11

Supplementary Table 3: The amino acid residue substitutions explored at the BWC0977 interaction sites, using mutagenesis experiments (*E. coli* MG1655). 12

Supplementary Table 4. Antibacterial effect of test compounds on *A. baumannii* ATCC 19606 resistant mutant colonies (mutant 1-5) isolated at 4x MIC (0.6 µg/mL) of BWC0977. 13

Supplementary Table 5: BWC0977 is superior to gepotidacin against global diverse clinical isolates. 14

Uncropped gel images 15

Supplementary information references 26

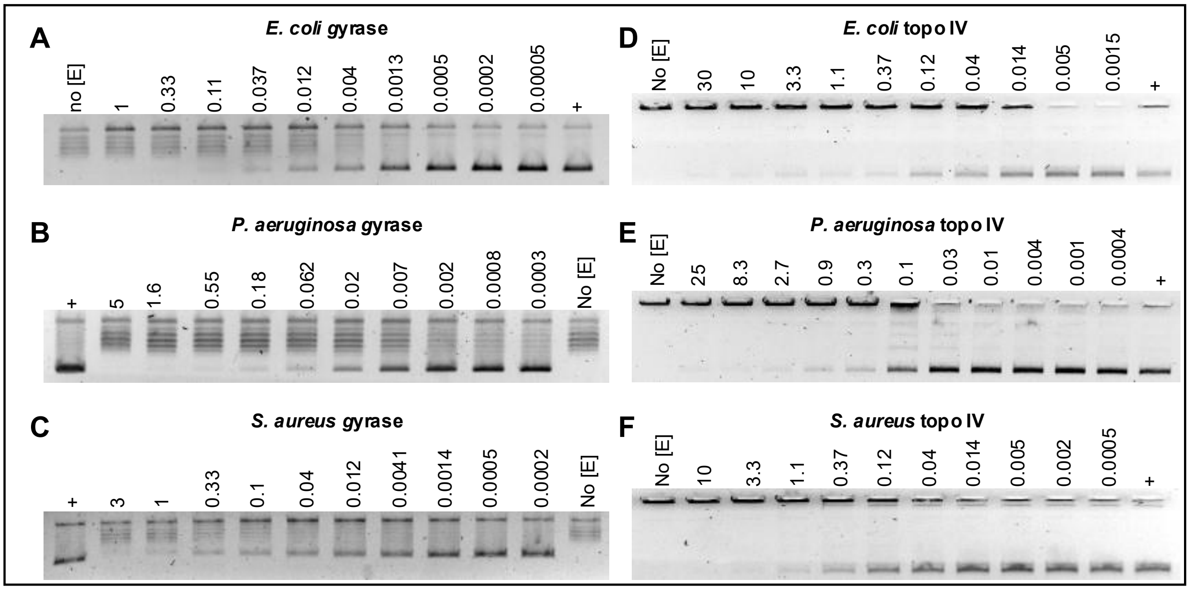

G

	IC_{50} in μM					
	E. coli		*P. aeruginosa*		*S. aureus*	
**Compound**	**gyrase**	**topo IV**	**gyrase**	**topo IV**	**gyrase**	**topo IV**
BWC0977	0.004± 0.001	0.013 ± 0.004	0.0094± 0.004	0.077± 0.002	0.009± 0.001	0.11± 0.027
Gepotidacin	0.77 ± 0.2 (0.34[1])	0.78 ± 0.3 (0.32)[1]	0.35	1.56	0.73 (0.047[2])	0.51
Compound 18c	0.1[3]	0.16[3]	ND	ND	ND	ND
Ciprofloxacin	0.26 ± 0.02	16 ± 3.1	0.213± 0.048	31.2	50±18	6.6±0.87

Supplementary Fig 1. BWC0977 inhibition on *E. coli*, *P. aeruginosa* & *S. aureus* gyrase and topoisomerase IV enzyme activities.
The gel images show the effect of varying concentrations (μM) of BWC0977 on (A-C) supercoiling and (D-F) decatenation activities. The + and no [E] represent the maximum (no compound, 100 %) and minimum (no enzyme, 0 %) reaction controls, respectively.
(G). IC_{50} values of BWC0977 and ciprofloxacin with *E. coli* (n=3), *P. aeruginosa* (n=2) and *S. aureus* (n=3) gyrase and topoisomerase IV (topo IV) enzymes. ND = not determined. n= number of independent experiments.

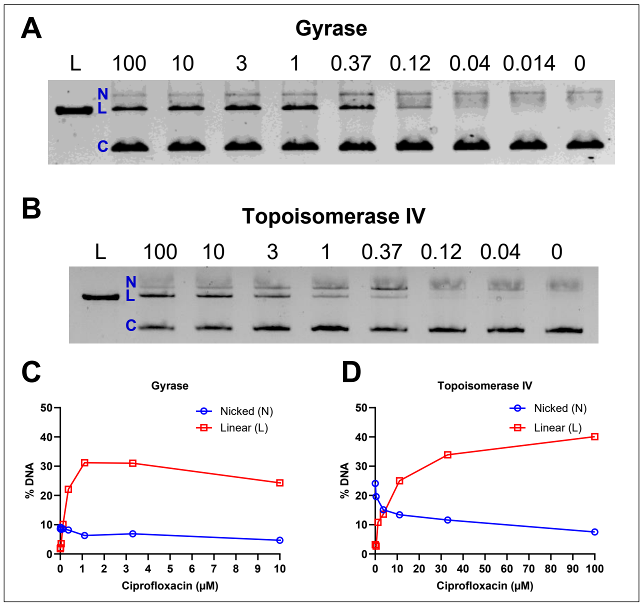

Supplementary Fig 2. Ciprofloxacin induces double-strand DNA breaks mediated by gyrase and topoisomerase IV enzymes.
The gel images show ciprofloxacin concentration (μM) response performed with (A) *E. coli* gyrase and (B) *E. coli* topoisomerase IV cleavage assays. The DNA bands on the gel images (L= linear DNA, N= nicked DNA, C= circular DNA) were quantified and plotted as % DNA induced by ciprofloxacin with (C) gyrase and (D) topoisomerase IV.

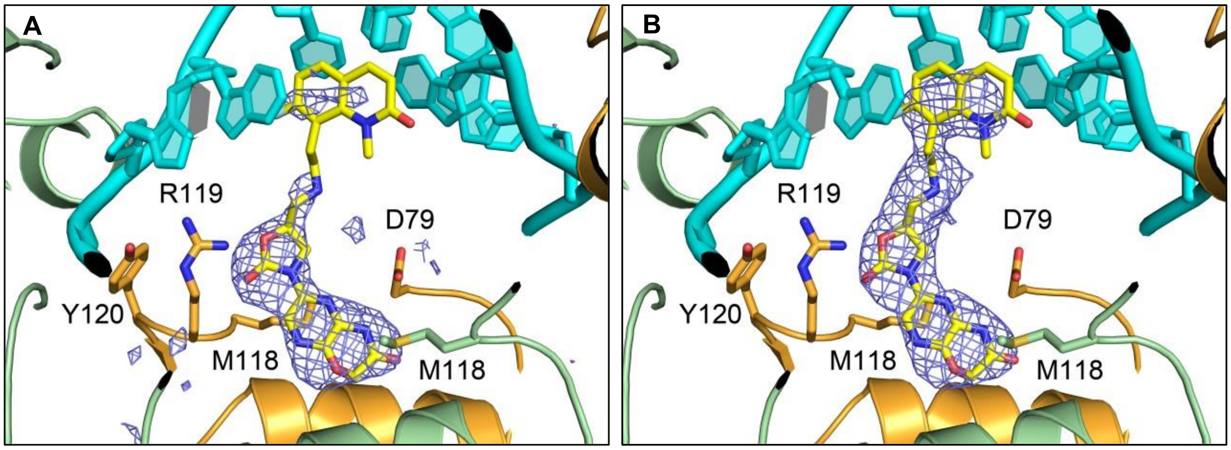

Supplementary Fig 3. Electron density maps from the 3.05 Å BWC0977 complex. OMIT and polder OMIT maps of the BWC0977 bound to the *K. pneumoniae* topoisomerase IV structure. (A) Conventional 2Fo–Fc OMIT map (blue mesh) contoured at 2.5 sigma, generated by omitting the ligand from the model prior to refinement. The map shows electron density consistent with ligand presence but includes potential bulk solvent effects. (B) Polder OMIT map (blue mesh) contoured at 5.0 sigma, calculated using phenix.polder to reduce bulk solvent flattening near the ligand. The map reveals enhanced and more continuous density for the ligand, supporting its accurate placement and occupancy in the binding site.

Each monomer (ParEC core) in the functional dimer is represented in orange and green colors. BWC0977 is shown in CPK representation with the position of the C, N, O and F atoms in yellow, blue, red and light green and DNA as cartoon in cyan.

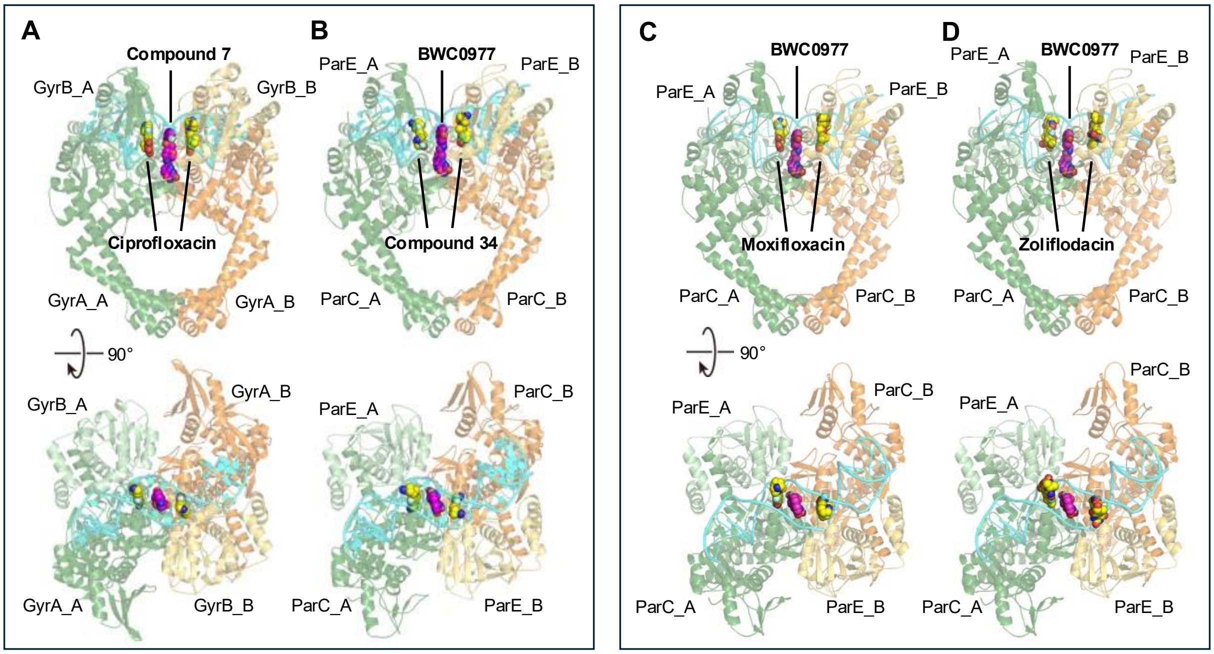

Supplementary Fig 4. Binding mode of ciprofloxacin, moxifloxacin, zoliflodacin and NBTIs shown with gyrase and topoisomerase IV models.
A single NBTI (compound 7, BWC0977) binds between the two cleavage sites at the dimer interface in both enzymes, whereas two molecules of ciprofloxacin, compound 34, moxifloxacin, or zoliflodacin bind at the DNA cleavage sites. In the models shown here, (A) *S. aureus* gyrase in complex with Compound 7 (PDB 5BS3) was superimposed on *Mycobacterium tuberculosis* gyrase in complex with ciprofloxacin (PDB 5BTC). (B) *K. pneumoniae* topoisomerase IV in complex with BWC0977 (PDB 9KGT) was superimposed with *K. pneumoniae* topoisomerase IV in complex with compound 34 (PDB 6WAA). (C) *K. pneumoniae* topoisomerase IV structure in complex with BWC0977 (PDB 9KGT) was superimposed with *A. baumannii* topoisomerase IV in complex with moxifloxacin (PDB 2XKK). (D) *K. pneumoniae* topoisomerase IV-BWC0977 structure was superimposed on *S. aureus* gyrase in complex with zoliflodacin (PDB 8BP2). Ciprofloxacin, compound 34, moxifloxacin and zoliflodacin molecules are shown as yellow spheres. The NBTIs, compound 7 and BWC0977 are shown as magenta spheres. Cartoon representations of the PDB structures 5BTC, 6WAA, 2XKK, and 8BP2 were omitted for clarity.

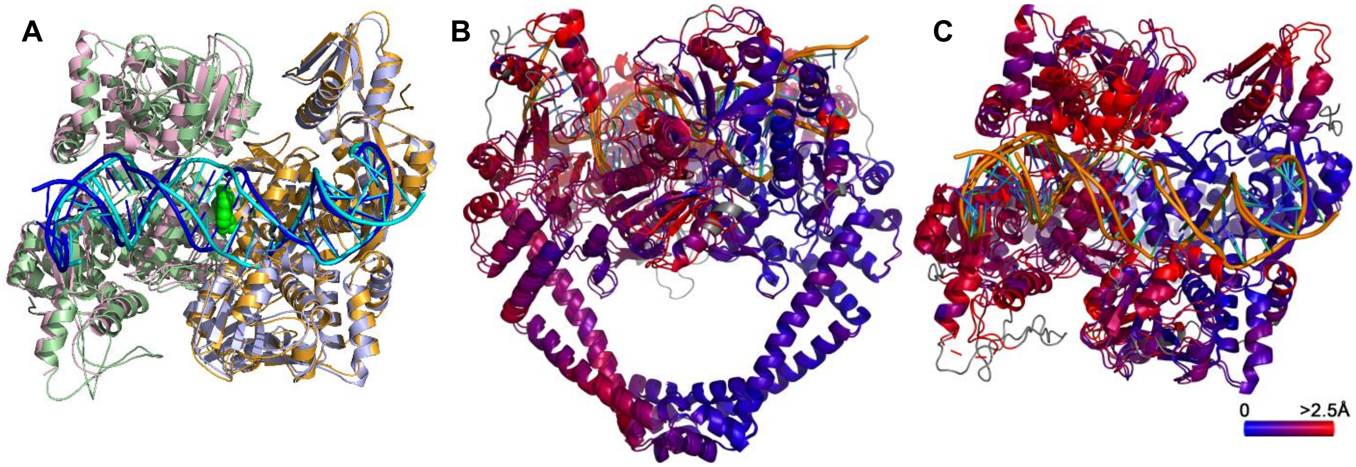

D

		ParEC-core (9KGT, this study)			
	Chain	A	B	C	D
ParEC-core (6WAA)	B	1.35	1.46	2.04	1.98
	D	1.34	1.24	1.24	1.33
	F	1.41	1.49	2.07	2.03
	H	1.31	1.20	1.24	1.29

Supplementary Fig 5. Structural comparison of ParEC-core complex with doubly nicked DNA duplex and compound 34.

(A) Superimposed view of the ternary complex of BWC0977 (ParEC dimer-BWC0977-duplex DNA), PDB: 9KGT and the ternary complex of compound 34 (ParEC dimer-compound 34-doubly-nicked DNA), PDB: 6WAA viewed down the twofold axis from ATP gate side. Bound BWC0977 is shown in the green CPK model in the middle of the figure, and the same color codes are used as in Fig. 3 (pale orange and pale green for ParEC-core, and the DNA backbone is cyan). The ParEC-core complexed with compound 34 is colored pale blue and pale magenta, and the doubly-nicked DNA backbone is depicted in blue. Molecular superposition is performed for the right-hand pair of the complexes in the figure (9KGT_chain A: pale orange and 6WAA_chain B: pale blue).

(B) and (C) Models are colored according to the superimposed Cα RMSD values of the corresponding locations. After superimposing on the right pair of functional dimers (9KGT_chain A vs. 6WAA_chain B), the RMSD of the entire dimer was calculated and colored according to its value. A scale bar showing the color gradient with corresponding RMSD values is shown. The figure was painted using ColorByRMSD, a Python script that functions on Pymol (Shandilya, S., Vertrees, J., Holder, T., https://pymolwiki.org/index.php/ColorByRMSD).

(D) Structural comparison among the four ParEC-core fused complexes in the published structure (PDB: 6WAA) and this study (PDB: 9KGT).

The RMSD values in Å were calculated between aligned pairs of the backbone Cα atoms in each monomer by GESAMT[4] in the CCP4 program suite[5]. AC and BD molecule pairs form ParEC-core dimers in our structure (9KGT), and BD and FH molecule pairs form ParEC-core dimers in the published structure (6WAA).

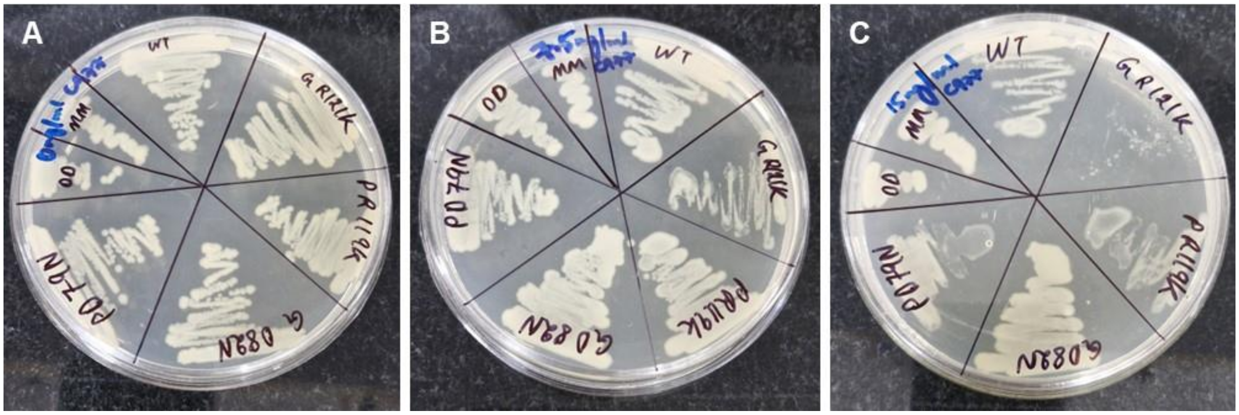

Supplementary Fig 6: Fitness of *E. coli* MG1655 WT and target mutant strains treated with BWC0977 in agar MIC experiment.

A dense bacterial culture of WT (wildtype), G R121K (GyrA R121K), P R119K (ParC R119K), G D82N (GyrA D82N), P D79N (ParC D79N), DD (GyrA D82N+ParC D79N), MM (GyrA M120A+ParC M118A) were diluted 1000-fold in Luria-Bertani broth. An equal volume of all strains were streaked on to the LB agar plates containing different concentrations of BWC0977 (A: 0 ng/mL, B: 7.5 ng/mL, C: 15 ng/mL) and the petriplates were incubated overnight at 37°C for the colonies to grow.

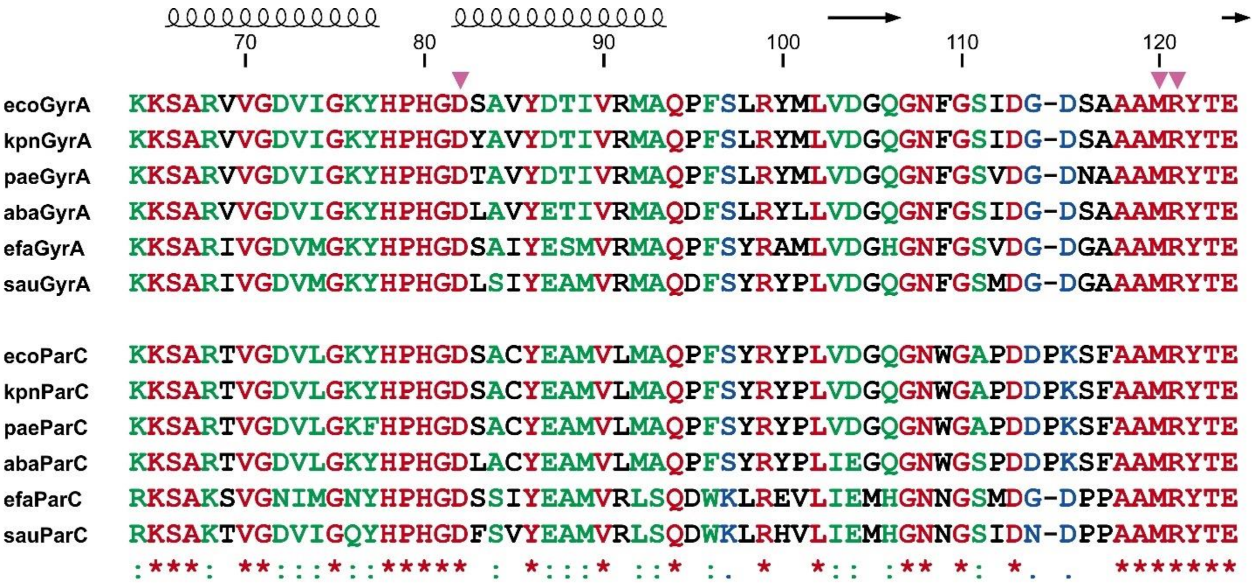

Supplementary Fig 7. Conservation of BWC0977 binding pocket (GyrA and ParC) residues. The amino acid sequences of GyrA and ParC from *E. coli* (*eco*), *K. pneumoniae* (*kpn*), *P. aeruginosa* (*pae*), *A. baumannii* (*aba*), *E. faecalis* (*efa*) and *S. aureus* (*sau*) are aligned with ClustalW multiple sequence alignment. Letters in red, green and blue are identical, strongly similar and weakly similar amino acids, respectively. The residue number corresponds to that of *eco*GyrA. The secondary structure elements of *kpn*ParC are shown on the top as coils and arrows for α-helices and the β-strands, respectively. Magenta triangles indicate the three residues important for BWC0977 binding (D82, M120 and R121 of *eco*GyrA, D79, M118 and R119 of *kpn*ParC).

Supplementary Table 1. Structural comparison among the four ParEC-core complexes in the two functional dimers in the asymmetric unit.

Chain	A	B	C	D
A	-	1.15	1.64	1.68
B		-	1.43	1.40
C			-	1.03
D				-

The RMSD values in Å were calculated between aligned pairs of the backbone Cα atoms in each monomer by GESAMT (ref) in the CCP4 program suite. AC and BD molecule pairs form functional dimers.

Supplementary Table 2. BWC0977 interactions important for topoisomerase IV (ParEC) inhibition.

Residue	Chain	Atom		Residue	Chain	Atom	distance (Å)
pyrazino-oxazinone and oxazolidinone moieties side (Fig. 3)							
M118	D	SD	-	BWC0977	J	Ring A	3.9
M118	B	SD	-	BWC0977	J	Ring B	3.1
D79	D	OD2	-	BWC0977	J	N26	3.3
D79	D	OD2	-	BWC0977	J	N22	2.9
D79	B	OD2	-	BWC0977	J	N32	3.4
R119	D	OD1	-	BWC0977	J	O20	2.8
R119	D	NH1	-	BWC0977	J	O34	2.9
R119	D	N	-	BWC0977	J	O34	3.5
R119	D	NH1	-	D79	D	OD1	3.7
R119	D	NH2	-	Y120	D	OH	3.3
R119	D	NE	-	Y120	D	OH	2.9
Y120	D	OH	-	DNA-G12	G	P	3.0
7-Fluoro-1-methylquinolin-2(1H)-one moieties side (Fig. 4)							
DNA-T13	H	Ring	-	BWC0977	J	Ring C	4.1
DNA-A13	H	Ring	-	BWC0977	J	Ring C	3.8
DNA-T13	G	Ring	-	BWC0977	J	Ring D	3.8
DNA-T14	G	Ring	-	BWC0977	J	Ring D	3.9
DNA-A13	H	N9	-	BWC0977	J	F12	3.3
DNA-A13	H	N7	-	BWC0977	J	F12	3.5
DNA-A13	G	N9	-	BWC0977	J	Ring D	3.4

Bond length of all the polar interactions, aromatic–aromatic interactions, and sulfur-aromatic interactions shown in Fig. 3 and 4, which are depicted in dotted lines. Measured lengths were rounded down to one decimal place.

Supplementary Table 3: The amino acid residue substitutions explored at the BWC0977 interaction sites, using mutagenesis experiments (*E. coli* MG1655).

target subunit		Comment
GyrA	ParC	
M120K	M118K	Synthetically lethal - nonviable
M120V	M118V	Speculated to be nonviable
M120A	M118A	viable – MIC tested
R121K	WT	viable – MIC tested
WT	R119K	viable – MIC tested
R121K	R119K	Synthetically lethal – nonviable
R121A	WT	Synthetically lethal – nonviable

The table lists the amino acid substitutions explored at the BWC0977 interacting M120 and R121 residues in *E. coli* MG1655 and the experimental outcomes.

Supplementary Table 4. Antibacterial effect of test compounds on *A. baumannii* ATCC 19606 resistant mutant colonies (mutant 1-5) isolated at 4x MIC (0.6 µg/mL) of BWC0977.

MIC_{90} in (µg/mL)		Resistant mutant colonies of BWC0977				
**Compounds**	***A. baumannii* ATCC 19606 (WT)**	**1**	**2**	**3**	**4**	**5**
BWC0977	0.15	0.6	0.07	0.07	0.07	0.3
Ciprofloxacin	0.6	2.5	2.5	2.5	2.5	2.5
Moxifloxacin	0.3	0.3	0.3	0.3	0.3	0.3
Novobiocin	10	10	10	10	10	10
Rifampicin	2.5	1.25	2.5	2.5	2.5	2.5
Solithromycin	2.5	5	2.5	2.5	5	2.5
Tetracycline	0.3	0.6	0.6	0.6	0.6	0.6
Meropenem	0.3	0.3	0.3	0.15	0.3	0.6

The table shows the antibacterial effect (MIC) of a few comparator antibiotics with multiple mode of action on BWC0977 resistant mutants in *A. baumannii* ATCC 19606 (mutant 1-5).

Supplementary Table 5: BWC0977 is superior to gepotidacin against global diverse clinical isolates.

MIC$_{90}$ (µg/ml) against diverse global isolates			
**Global clinical isolates**	**BWC0977**[6]	**Gepotidacin**[7,8]	**Ciprofloxacin**[6]
K. pneumoniae	2 (300[6])	16 (109[7])	> 32 (300[6])
E. faecium[6] *or faecalis*[7]	0.12 (126[6])	2 (107[7])	ND
S. aureus	0.03 (560[6])	0.5 (50[8])	ND
E. cloacae	2 (152[6])	16 (107[7])	ND
E. coli	0.5 (300[6])	4 (25[8])	ND
A. baumannii	1 (298[6])		> 32 (298[6])
P. aeruginosa	1 (300[6])		16 (300[6])

MIC$_{90}$ values not determined on identical panels of isolates. Data compiled from multiple reports. MIC$_{90}$ values of global collection of Gram-negative drug-resistant clinical isolates reported for ciprofloxacin and BWC0977 (except for *E. faecium* and *S. aureus,* the values are from diverse global isolates).
Number of isolates are mentioned in the parenthesis.

Uncropped gel images

Fig 2D
Fig 2E
Supplementary Fig 1A
Supplementary Fig 1B
Supplementary Fig 1C
Supplementary Fig 1D
Supplementary Fig 1E
Supplementary Fig 1F
Supplementary Fig 2A
Supplementary Fig 2B

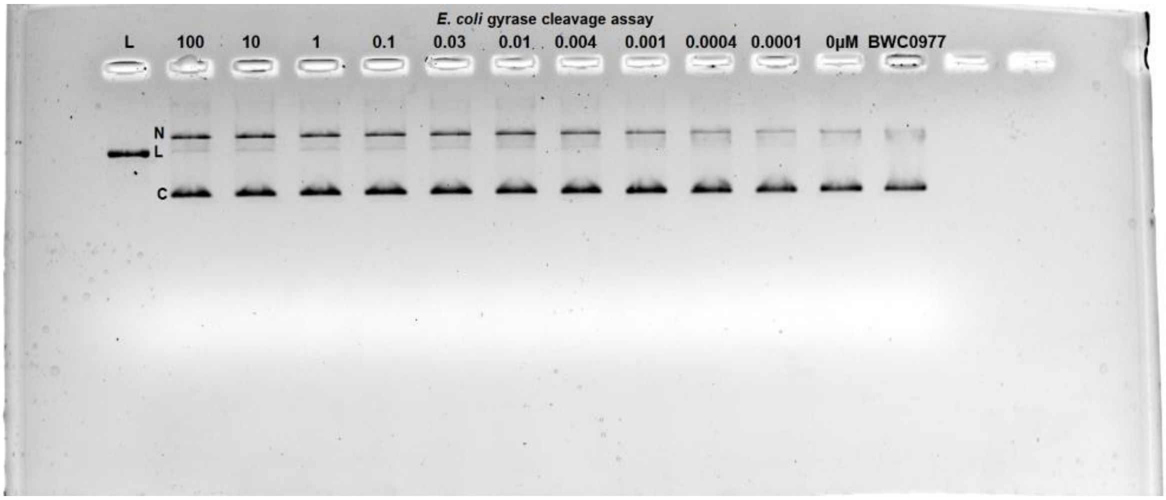

Fig 2D. *E. coli* gyrase cleavage assay in the presence of BWC0977.
The gel images show BWC0977 concentration response (μM) performed with *E. coli* gyrase in a DNA cleavage assay setup. L=linear DNA, N=nicked DNA, C=circular DNA.

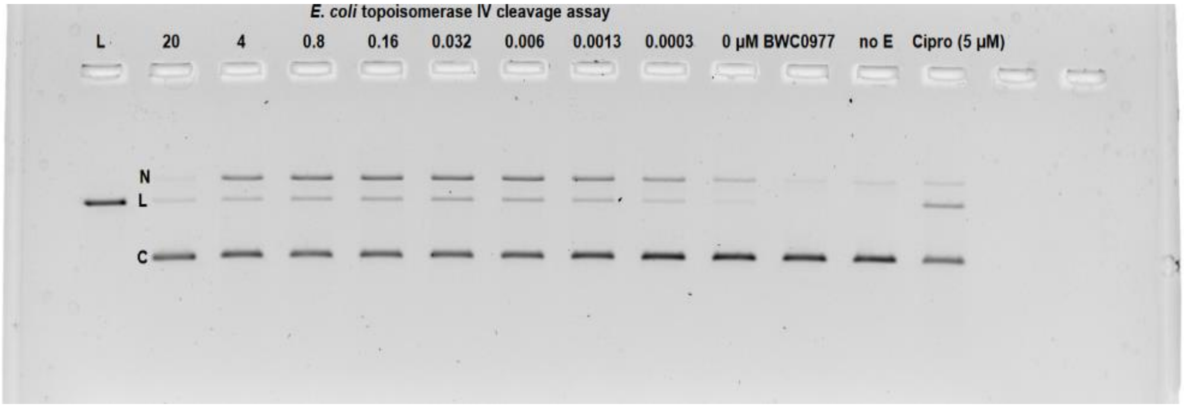

Fig 2E. ***E. coli* topoisomerase IV cleavage assay in the presence of BWC0977.**
The gel images show BWC0977 concentration response (μM) performed with *E. coli* topoisomerase IV in a DNA cleavage assay setup. L=linear DNA, N=nicked DNA, C=circular DNA.

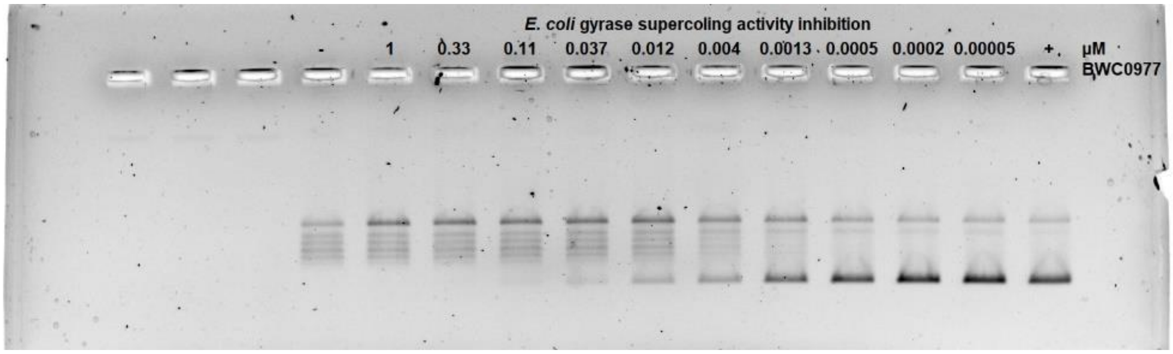

Supplementary Fig 1A. BWC0977 inhibition on *E. coli* gyrase enzyme activity.
The gel images show the effect of varying concentrations (μM) of BWC0977 on *E. coli* gyrase supercoiling activity. The + and − represent the maximum (no compound, 100 %) and minimum (no enzyme, 0 %) reaction controls, respectively.

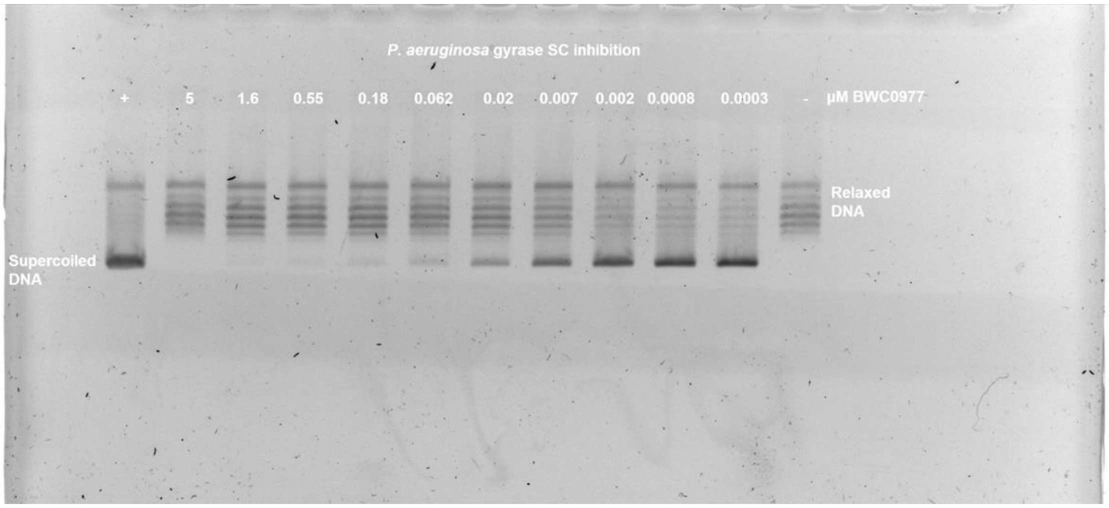

Supplementary Fig 1B. BWC0977 inhibition on *P. aeruginosa* gyrase enzyme activity. The gel images show the effect of varying concentrations (µM) of BWC0977 on *P. aeruginosa* supercoiling activity. The + and − represent the maximum (no compound, 100 %) and minimum (no enzyme, 0 %) reaction controls, respectively.

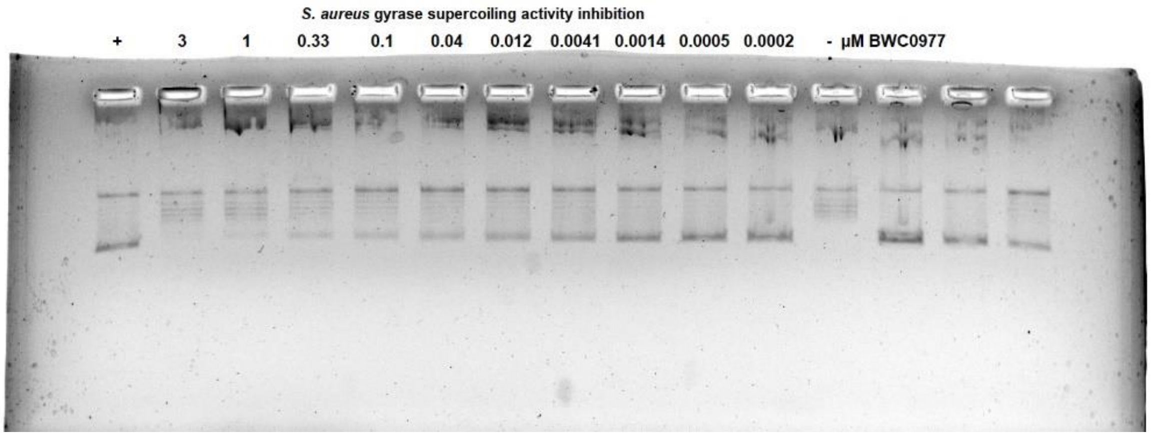

Supplementary Fig 1C. BWC0977 inhibition on *S. aureus* gyrase enzyme activity. The gel images show the effect of varying concentrations (µM) of BWC0977 on *S. aureus* supercoiling activity. The + and − represent the maximum (no compound, 100 %) and minimum (no enzyme, 0 %) reaction controls, respectively.

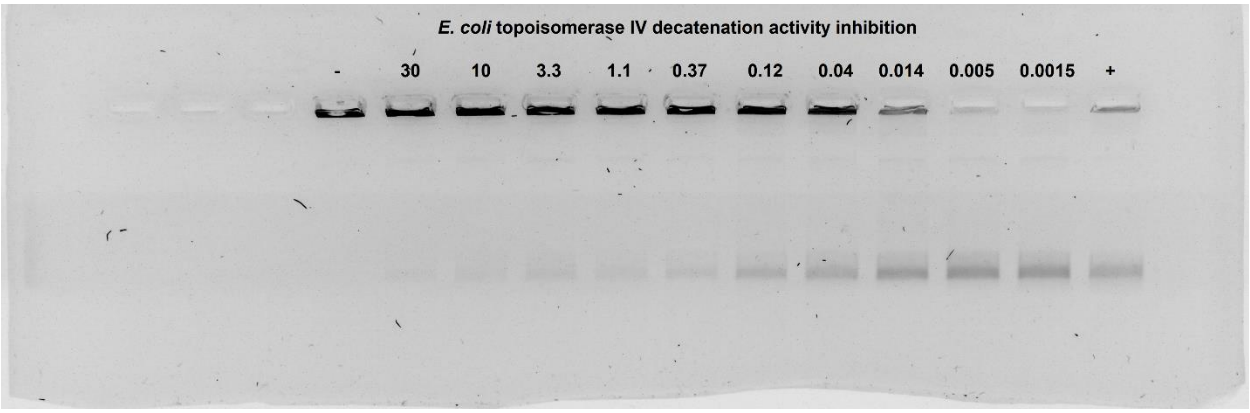

Supplementary Fig 1D. BWC0977 inhibition on *E. coli* topoisomerase IV enzyme activity. The gel images show the effect of varying concentrations (μM) of BWC0977 on *E. coli* topoisomerase IV decatenation activity. The + and − represent the maximum (no compound, 100 %) and minimum (no enzyme, 0 %) reaction controls, respectively.

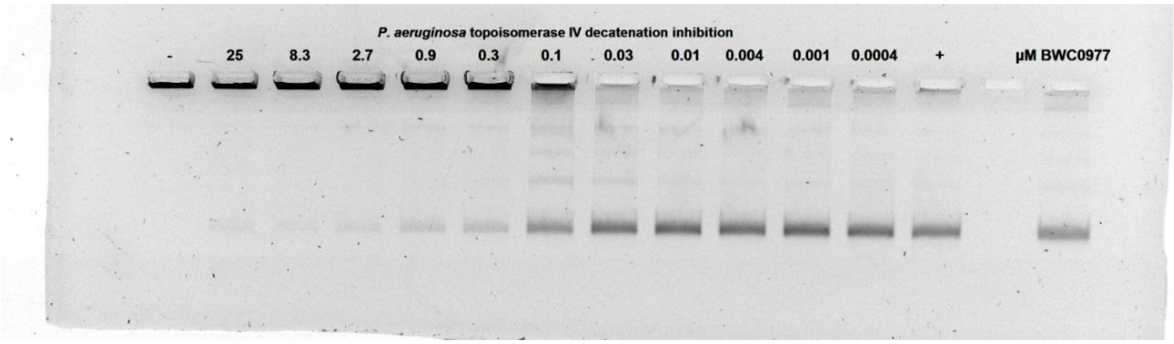

Supplementary Fig 1E. BWC0977 inhibition on *P. aeruginosa* topoisomerase IV enzyme activity.

The gel images show the effect of varying concentrations (µM) of BWC0977 on *P. aeruginosa* topoisomerase IV decatenation activity. The + and − represent the maximum (no compound, 100 %) and minimum (no enzyme, 0 %) reaction controls, respectively.

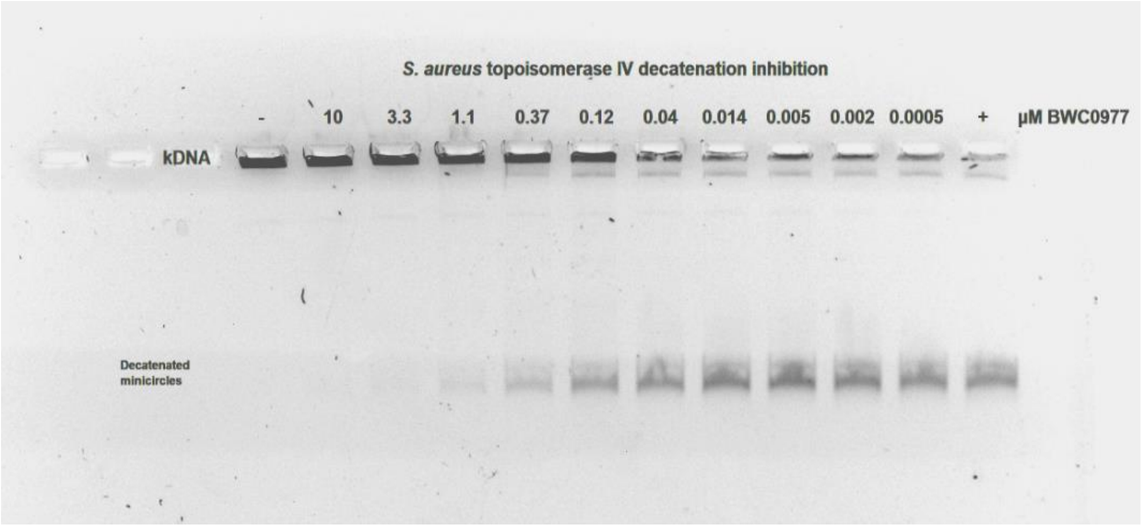

Supplementary Fig 1F. BWC0977 inhibition on *S. aureus* topoisomerase IV enzyme activity.
The gel images show the effect of varying concentrations (µM) of BWC0977 on *S. aureus* topoisomerase IV decatenation activity. The + and − represent the maximum (no compound, 100 %) and minimum (no enzyme, 0 %) reaction controls, respectively.

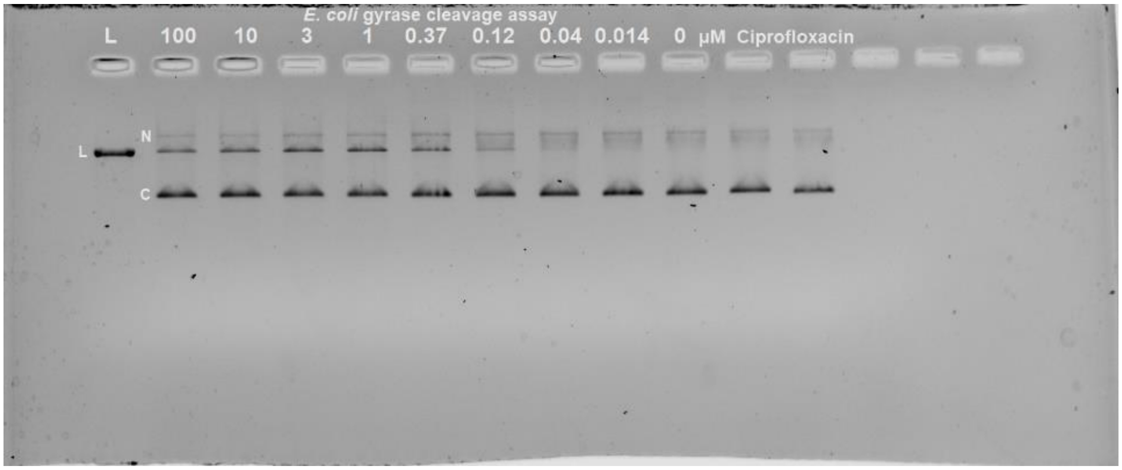

Supplementary Fig 2A. *E. coli* gyrase cleavage assay in the presence of ciprofloxacin. The gel images show ciprofloxacin concentration (µM) response performed with *E. coli* gyrase in cleavage assays. L= linear DNA, N= nicked DNA, C= circular DNA.

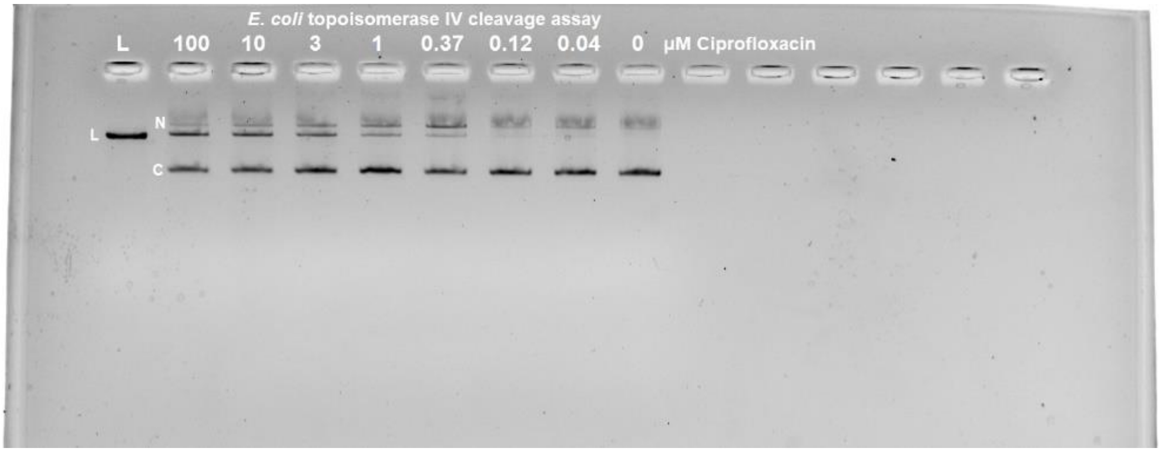

Supplementary Fig 2B. *E. coli* topoisomerase IV cleavage assay in the presence of ciprofloxacin.

The gel images show ciprofloxacin concentration (μM) response performed with *E. coli* topoisomerase IV cleavage assays. L= linear DNA, N= nicked DNA, C= circular DNA.

Supplementary information references

1. Oviatt, A. A. *et al.* Interactions between Gepotidacin and Escherichia coli Gyrase and Topoisomerase IV: Genetic and Biochemical Evidence for Well-Balanced Dual-Targeting. *ACS Infect. Dis.* **10**, 1137–1151 (2024).
2. Gibson, E. G., Bax, B., Chan, P. F. & Osheroff, N. Mechanistic and Structural Basis for the Actions of the Antibacterial Gepotidacin against Staphylococcus aureus Gyrase. *ACS Infect. Dis.* **5**, 570–581 (2019).
3. Cumming, J. G. *et al.* Discovery of a Series of Indane-Containing NBTIs with Activity against Multidrug-Resistant Gram-Negative Pathogens. *ACS Med. Chem. Lett.* **14**, 993–998 (2023).
4. Krissinel, E. Enhanced fold recognition using efficient short fragment clustering. *J. Mol. Biochem.* **1**, 76–85 (2012).
5. Agirre, J. *et al.* The CCP4 suite: integrative software for macromolecular crystallography. *Acta Crystallogr. Sect. Struct. Biol.* **79**, 449–461 (2023).
6. Hameed P, S. *et al.* BWC0977, a broad-spectrum antibacterial clinical candidate to treat multidrug resistant infections. *Nat. Commun.* **15**, 8202 (2024).
7. Arends, S. J. R. *et al.* 701. Comparison of MIC Results for Gepotidacin by Agar Dilution and Broth Microdilution Methods. *Open Forum Infect. Dis.* **6**, S317 (2019).
8. Flamm, R. K., Farrell, D. J., Rhomberg, P. R., Scangarella-Oman, N. E. & Sader, H. S. Gepotidacin (GSK2140944) In Vitro Activity against Gram-Positive and Gram-Negative Bacteria. *Antimicrob. Agents Chemother.* **61**, e00468-17 (2017).
