# Supplementary material for: Structural interactions of BWC0977 with Klebsiella pneumoniae topoisomerase IV and biochemical basis of its broad-spectrum activity
Source: Commun Biol. 2025 Nov 25;8:1666. doi: 10.1038/s42003-025-09055-y (PMC12647645; doi:10.1038/s42003-025-09055-y)
Supplement: Supplementary file 3 — Description of Additional Supplementary Files [file 42003_2025_9055_MOESM3_ESM.pdf]

## Description of Additional Supplementary files

**File name:** Supplementary Data

**Description:** The source data behind the graphs in the paper
